# Factors influencing access to primary health care in Luanda, Angola

**DOI:** 10.1186/s12913-024-12120-7

**Published:** 2025-02-14

**Authors:** Antónia Sónia de Carvalho Maquengo, Alfredo Manuel Bastos, Marli Stela Santana

**Affiliations:** 1 Catholic University of Angola, Higher Institute of Health Sciences, Pedro de Castro Van Dúnen Avenue, 24, PO Box 2064, Luanda, Angola; 2 Interdisciplinary Studies and Research Center at Catholic, University of Angola, Pedro de Castro Van Dúnen Avenue, 24, PO Box 2064, Luanda, Angola; 3 Scientific Initiation Program, Catholic University of Angola, Pedro de Castro Van Dúnen Avenue, 24, PO Box 2064, Luanda, Angola

**Keywords:** Adherence, Satisfaction, Primary health care

## Abstract

**Introduction:**

The primary health care system is the first of the three hierarchical levels of care, and it is the first point of contact of the population with the health system. In Luanda, Angola, the primary care network presents challenges in its management, as well as in the perception of its purposes, which constitutes a serious problem for the demand for health services, being the reason for this study. The present research aimed to evaluate the level of adherence and satisfaction related to the health care system and their influence on usage of the public primary health care facilities in Luanda, from the perspective of health professionals and users.

**Methods:**

A cross-sectional and descriptive study was carried out on 120 health professionals and 423 users.

**Results:**

We found a statistically significant association between the level of education and the professionals' disbelief in health services (*P* = 0.001), as well as the users' family income (*P* = 0*.*0002).

**Discussion:**

The users' perception is that there is a switch in test results (*P* = 0.01). Furthermore, they also believe that when attending health units, the user leaves sicker than he entered (*P* = 0.01).

**Conclusion:**

Thus, it was found that the level of user adherence to the services of the primary health care units in Luanda ranged between "good" and "acceptable". We identified elements capable of compromising the quality of services and, consequently, interfering with adherence to them, which suggests the need for the development of management strategies for health facilities, in order to overcome the challenges presented in the study.

**Supplementary Information:**

The online version contains supplementary material available at 10.1186/s12913-024-12120-7.

## Introduction

Sub-Saharan Africa has faced difficulties in developing, representing a region of the world with serious public health problems, high rates of infant and maternal mortality, and infectious diseases, which demonstrate and contribute to the low rate of human development [1]. Despite economic growth, sub-Saharan African countries still have social inequalities where few people have access to goods and social infrastructure, such as the National Health System. The majority of the population still live below the poverty line, especially those who live in rural areas, which make up the majority [1].

The National Health System is made up of all public and private entities that carry out promotion, prevention, and treatment activities in the health area [2]. The Angolan National Health Service constitutes the largest health network in the country with a focus on Primary Health Care (PHC). From 1992 onwards, the provision of healthcare was no longer the exclusive preserve of the state with the legalization of the private sector. Individual participation in healthcare costs was also introduced during this period, thus transforming the generally free and universal system [3].

The health care delivery network has a current health center ratio of 1 to 20,000 inhabitants, which suggests that there is a huge lack of basic health services to meet the needs of the population [3]. At the level of PHC, preventive and curative activities for common illnesses and injuries are implemented, such as health education, pre- and post-natal consultations, family planning, birth assistance and basic and complete obstetric care, vaccination, and control of the child's development and growth [4].

The public health system in Angola still suffers from insufficient human resources, lack of basic sanitation, low coverage of PHC, and poor functioning a factor that causes overcrowding in medical emergencies in hospitals with a polyvalent or specialized level of care [5]. Luanda province has a health network made up of 156 public health units for PHC, including 10 municipal hospitals, 30 health centers of reference, 37 health centers, 29 type II health posts, and 50 type I, divided by municipality or district region [6].

In the field of health, countries like Angola face great challenges in the management of health services [7], and the assessment of their quality and satisfaction levels is an indispensable element for the proper management of resources, promoting the importance of the user in the health system.

User satisfaction can be seen as a personal evaluation of health care services and their providers. It serves as a link between the services provided and the needs and wishes of the users, and allows for an evaluation that directly reflects the users' perspective of a service. The data related to user satisfaction can serve as an indicator of the quality of the service provided, thus promoting its use [8].

User satisfaction is related to the quality of the services that are provided. The user is said to be satisfied when their experience of interacting with the service has exceeded their expectations [9].

However, to assess user satisfaction regarding the quality of services, two approaches should be considered, where one reports the experience of users in relation to the services offered, and the other seeks to estimate the level of satisfaction of users that is considered a reflection of their personal preferences, perceptions, and expectations, i.e., to identify the elements that, in their opinion, help define what quality service is [10].

Health systems that have robust PHC programs are more efficient. Health systems with a more comprehensive and integral primary care network are more effective, as they bring better results and better application of health resources. For this reason, discussing the financing of a PHC network is crucial to the needs for an efficient health system [11].

In Angola, the primary care network presents challenges in its management, as well as in the perception of its purposes, which constitutes a problem for the demand for health services. It is in this context that the possibility of conducting a study with the subject to evaluate the level of adherence and satisfaction of health professionals and users with regards to PHC and the influence of professionals and users in promoting access to the facilities in Luanda, Angola.

## Methodology

### Study design

A cross-sectional and descriptive study was conducted in two primary health care units (PHCUs) of the public health sector in Luanda, the capital of Angola, with 364 health professionals and 8.000 users seen in the two health facilities.

### Study area

The study was carried out in two health units in Luanda: the Palanca Health and Maternity Center located in the municipality of Kilamba Kiaxi, and the Terra Nova Reference Health Center located in the municipality of Luanda, Urban District of Rangel.

### Sample calculation

The calculation of the sample of health professionals took into account the considerations of Luiz and Magnanini [12] and the frequency described by data issued by the statistical sector of the studied health units, based on an estimated frequency of 50% (of health professionals) and 60% (of users) for what we intended to study. The study was structured with a confidence level of 95% and margin of error of 5%, as well as with an additional 20% on the total sample to repair losses and to increase its significance.

### Sample selection

A total of 120 health professionals and 423 users, aged ≥ 18 years, of both genders (and in the case of health professionals, those in active work) were selected in probabilistically in order of arrival, intentional way with direct contact from the researcher.

### Data collection

The data were collected through two questionnaires developed for this study (supplementary file), one for health professionals with 22 questions, and one for the users which had 32 questions. Both were structured with questions organized in four parts: socioeconomic and demographic data of the respondents; the professionals' assessment of the working conditions and the reasons for adhering to the services offered in the health unit; the users' satisfaction concerning the services, professional performance, and facilities; and the identification of the weak points of the health unit by the users, with some questions allowing the respondent to choose more than one option as an answer.

The questionnaire was validated in a simple and unprofessional way to allow for adjustments, which included removing irrelevant information and duplicities, in a pilot study conducted with 20 people before the present study. The questionnaire was filled out in Portuguese, taking into account other local languages commonly spoken in Luanda Kimbundo, Umbundo, and Lingala in a maximum time of 20 min. Data collection occurred from November 2021 to March 2022.

### Data management and analysis

The information from the questionnaire was stored in a Excel database, and descriptive and inferential statistical analyses were performed using Epi info™ software. 7.2.2.6 (Centers for Disease Control and Prevention—CDC, Atlanta, GA, USA). The significance level adopted was *P* ≤ *0*.05.

### Ethical approval and consent to participate of the study

The project was evaluated by the Ethics Committee on Human Research of the Catholic University of Angola and approved under protocol n. 76/2020, to safeguard the rights and dignity of the research subjects. Participation in the research was voluntary, and those who participated in the research signed an informed consent form as a sign of acceptance.

## Results

Sociodemographic data of the 120 health professionals are described in Table 1; 66.6% were female, with a greater predominance in the 32 to 45 age range (46.6%) and having a higher level of education (67.5%). According to the data collected, concerning the users, there was a higher participation of individuals aged between 18 to 30 years (63.36%), with a higher frequency of females (83.92%), in which the majority of participants had a secondary level of education (67.14%) and a family income below 118.57 USD (Table 1).

**Table 1:** Characterization of the socioeconomic and demographic profile of the research participants

	Professionals		Users	
Variables	*N* = 120	%	*N* = 423	%
Gender				
Female	80	66.6	355	83.92
Male	40	33.3	68	16.08
Age				
18 to 30	25	20.8	268	63.36
31 to 45	56	46.6	125	29.54
46 to 65	39	32.5	30	7.12
Education level				
Illiterate	0	0	5	1.18
Primary	0	0	45	10.64
Secondary	39	32.5	284	67.14
Higher education	81	67.5	89	21.04
Monthly family income^a^	-	-		
< 118.57 USD^b^	-	-	272	64.30
> 118.57 USD	-	-	151	35.7

^a^The question about the income of the professionals was removed from the questionnaire because they did not want to answer it.

^b^It is equivalent to less than 60 thousand kwanzas (kz), the Angolan currency

### Socioeconomic and demographic characteristics of participants

Table 2 represents the working conditions of health professionals and the structure of the health unit. Forty-six percent of respondents have between 1 to 10 years of experience working in the health sector; 50% say they work at an intense pace; and 58.33% consider communication with users to be easy.

**Table 2:** Professional’s evaluation of the working conditions

Variables	*N* = 120	%
Time of work experience (in years)		
1 to 10	55	46
11 to 20	45	37
21 to 30	14	12
31 to 40	6	5
The pace of work in the PHCU		
Intense	60	50
Fair	54	45
Calm	6	5
Communication with patients has been		
Very Easy	25	20.83
Easy	70	58.33
Hard	20	16.67
They don't understand	5	4.17
The work provided is humanized		
Yes	83	69.17
No	13	10.83
Sometimes	24	20
There are shortages of medicines and other materials in the PHCU		
Yes	73	60.83
No	5	4.17
Sometimes	42	35
The service at PHCU is done by:		
Attendance sheet	48	40
Time of arrival	100	83.33
Appointment	11	9.17
Seriousness	74	61.67
Convenience	2	1.67
Alphabetical/numerical order	2	1.67
What should be improved in the PHCU		
Quality of services	81	67.5
Business hours	41	34.17
Sanitary Facilities	68	56.67
Information System	37	30.83
Equipment (furniture/clinical)	110	91.67
Other	44	36.67

Most professionals (60.83%) reported a shortage of medicines and other materials in the PHCUs and reported that the order of care is carried out by order of arrival (83.33%) and by severity (61.67%). The quality of services offered by the health unit (67.5%), the information system (30.83%), and the equipment (furniture/clinic) (91.67%) were noted to be the highest priorities in the improvement process.

### Characterization of working conditions and reasons for adhering to services offered in health units from the perspective of professionals

Table 3 describes the reasons why users adhere to the services offered in PHC facilities.

**Table 3:** Professionals' evaluation of the reasons for adhering to the services offered at the health unit

Variables	*N* = 120	%
What are the underlying causes of the high demand for secondary and tertiary level UCSP?		
Lack of PHCU facilities	55	45.83
Poor performance of primary-level	19	15.83
Lack of doctors at the primary-level	77	64.17
Few professionals	82	68.33
Nearness to the user's home	40	33.33
Exemption of payment in the rendering of services	34	28.33
Feel better served	42	35
Speed of service	29	24.17
Lack of knowledge of the users	97	80.83
The users adhere to the services provided in this PHCU because:		
It's close to home	66	55
Serves you well	107	89.17
No charge	75	62.5
Fast service	84	70
Users do not adhere to the services provided in this PHCU because:		
They don't believe in the services	11	9.17
They are not well received	14	11.67
Delayed service	34	28.33
It's far from home	65	54.17
They prefer private units	70	58.33
They prefer professionals who are their fellow countrymen	46	38.33
They don’t know anybody in the PHCU	9	7.5

According to health professionals, the high demand for secondary and tertiary-level health units is due to the lack of primary-level health units (45.83%). The poor performance of primary level health units (15.83%), the absence of doctors in these units (64.17), or the lack of knowledge of the users (80.83%) were also stated.

The professionals believe that the adherence to the services provided in the PHCUs is due to the proximity to the user's home (55%), the service being free of charge (62.50%), and the fast speed of service (62.5%).

The reasons for the non-adherence of users to the services provided in the PHCUs are the lack of credibility in the services (9.17%), the distance between the residence and the PHCUs (54.175%), and the preference for professionals who are fellow countrymen (38.33%).

### Satisfaction of service delivery by user

Regarding satisfaction with the distance between the residence and the PHCUs, 30.02% felt satisfied. In the evaluation of the physicians' performance, regarding diagnosis and medication, 22.22% rated it as good. Concerning nurses' diagnostic and medication performance, 81.56% of the participants rated it as good. Of the 423 participants in the study, 72.34% of our participants had never had contact with a doctor in the health units.

The participants rated the quality of health unit facilities as good (40.43%), the consultation office as acceptable (59.57%), the equipment (clinical furniture) as acceptable (62.17%), hygiene and cleanliness as poor (64.54%), and 38.30% rated the comfort as good (Table 4).

**Table 4:** User satisfaction regarding service, professional performance, and facilities

Variables	*N* = 423	%
Satisfaction with the distance to the PHCU		
Very satisfied	85	20.09
Not very satisfied	117	27.66
Not satisfied at all	93	21.99
Satisfied	127	30.02
Irrelevant	1	0.24
Evaluation of physician performance concerning diagnosis and medication		
Very good	2	0.47
Good	94	22.22
Acceptable	15	3.55
Sufficient	0	0
Bad	1	0.24
Very bad	5	1.18
Never had contact with a doctor	306	72.34
Nurses' performance evaluation concerning diagnosis and medication		
Very good	3	0.71
Good	345	81.56
Acceptable	67	15.84
Sufficient	0	0
Bad	0	0
Very bad	8	1.89
Assessment of the quality of the health unit facilities in terms of		
Visual signaling		
Very good	12	2.84
Good	171	40.43
Acceptable	203	47.99
Sufficient	2	0.47
Bad	7	1.65
Very bad	28	6.62
Doctor*'s* offices		
Very good	3	0.71
Good	152	35.93
Acceptable	252	59.57
Sufficient	0	0
Bad	0	0
Very bad	16	3.78
Equipment (clinical furniture)		
Very good	1	0.24
Good	61	14.42
Acceptable	263	62.17
Sufficient	0	0
Bad	3	0.71
Very bad	95	22.46
Hygiene and cleaning		
Very good	1	0.24
Good	22	5.20
Acceptable	110	26
Sufficient	0	0
Bad	17	4.02
Very bad	273	64.54
Comfort		
Very good	2	0.47
Good	162	38.30
Acceptable	123	29.08
Sufficient	1	0.24
Bad	3	0.71
Very bad	132	31.21

Regarding the weaknesses of the health units, the main complaints were about the lack of materials (92.43%), charges for public health services (29.53%), errors in test results (22.93%), and the lack of doctors (17.02%). Most of the participants (79.20%) resort to some sort of private health service when they have a serious problem.

In terms of user expectations in the improvement of the services provided, the participants elected those that are a priority for them: 37.12% for the opening hours, 46.68% for the waiting time for consultation, and 89.13% for lack of necessary materials (Table 5).

**Table 5:** Users' identification of the weaknesses of the health units

Variables	*N* = 423	%
Main complaints at PHCU		
Charging for services	125	29.53
Many people die	6	1.42
Errors in test results	97	22.93
Switch of test results	2	0.47
The person gets sicker	6	1.42
Lack of materials	391	92.43
It is overcrowded	235	55.56
Lack of doctors	72	17.02
What makes you use another health service		
There are no doctors in the PHCU	67	15.84
Poor service at PHCU	5	1.18
The seriousness of the disease	335	79.20
Other^a^	78	18.44
No difficulties	99	23.40
Improvement Expectations		
Quality of services	55	13
Opening time	157	37.12
Method of service	150	35.46
Waiting time for consultation	197	46.68
Lack of required materials	377	89.13
Performance of administrative staff	2	0.47
Performance of the medical and technical staff	52	12.29
Facilities	88	20.80
Information systems	0	0
Other^a^	146	34.52

^a^Laboratory, pharmacy, fast services, and humanization

### Statistical association between the independent variable and the dependent variables

There was a statistically significant association between the level of education of health professionals and the first-come, first-served (*P* = 0.005), and the disbelief in the services provided in the PHCUs (*P* = 0.001). Regarding users, there was a statistical association between the level of education and family income (*P* = 0.0002). The performance of nurses regarding diagnosis and medication (*P* = 0.0002), and the opinion that the person leaves sicker than when he/she entered the PHCUs (*P* = 0*.*01). Regarding the priorities for what needs to be changed in the health units, there was also a statistical association between the level of education of users and understanding of the way of caring (*P* = 0.03) (Table 6).

**Table 6:** Simple linear regression between the level of education and other variables

Variables/Professionals	*P* ≤ 0.05*
Attendance at PHCU is on a first-come, first-served basis	0.005
Because they do not believe in the services provided, users look for other health units (private or secondary level)	0.001
Variables/Users	
Monthly family income below < 118.57 USD (equals to 60,000Kz)	0.0002
Does not go to public health unit because there is switch of test results	0.01
Does not attend public health units because he believes the person gets sicker	0.01
Rates as good the performance of nurses regarding diagnosis and medication	0.0002
Rates the quality of safety signage as acceptable	0.04
Rates the quality of the equipment as acceptable	0.01
Rates the comfort of the infrastructure as good	0.01
The way of serving the user needs to be improved	0.03

^*^Significance level *P* ≤ 0.05*.* F-statistic and T-statistic were used in this linear regression

## Discussion

The results of this study showed that the level of education and family income are the main factors that influence access to primary health care in Luanda, as the majority of professionals had higher education (67.5%), which demonstrates that health professionals have increasingly invested in academic training, taking into account the various changes occurring in the Angolan health system [13]. It was also found that the majority of users had secondary education (67.14%), showing greater satisfaction with the services offered, but also with the capacity for critical evaluation, being capable of pointing out the weak points of the services [14].

From the perspective of health service professionals, the level of education has been shown to influence access to PHCUs. Thus, the higher the level of education of health professionals, the greater their ability to understand that the organization of the PHCS in Luanda can interfere with adherence to the services offered. On the other hand, from the users' perspective, individuals with higher education have lower satisfaction with the services offered, compared to users with basic education, as already described in Africa [15].

Users with a monthly family income of less than 118.57 USD are more likely to subscribe to the services offered by a PHCUs, but they complain about the shortage of necessary materials in offices, test laboratories, and above all, the shortage of medicines in pharmacies because most of the time, they have to resort to purchasing from commercial pharmacies, which represents a challenge for the low-income population and a limitation in access to treatment.

The health sector has a strong tendency for the hiring of women, possibly due to the professional activities of caring for the vulnerable and sick resembling traditional female daily life in Angola, where the practice of caring is something intrinsic to this gender, in a population where females represent 51% of the inhabitants [16]. Regarding the participation of female professionals in this study (66.6%), there was a similarity with studies conducted in Brazil and Cape Verde where women were also the majority of respondents [17–19, 13].

The present study had a higher participation of female users (83.92%) [20] than men, and according to Brazilian research [21], women use health services more often than men, corroborating the data from the present study. However, the present research showed a greater predominance of users aged 18 to 30 years (63.36%) different to the Brazilian research [22] which showed greater adherence of users aged 30 to 54 years (44.4%).

The working time is considered as the period a health professional is available for the execution of professional tasks, and has as a counterpart, most of the time, the basic wage [23]. In our research, almost half of the studied population (46%) had up to 10 years of work activity experience in the health area, which can be considered a positive aspect, considering that the longer the time of work activity, the greater the experience and ease of dealing with health problems [24].

Work conditions may influence the performance of the employees, where we found a significant number of participating professionals (16.67%) who were dissatisfied with the working conditions, stating that they were unfavorable (60.83%), alleging the shortage of medicines and other materials for the provision of services. These findings corroborate data from studies conducted in ten countries in four sub-regions of Africa (Central, Eastern, Southern, and Western), as well as Portuguese-speaking African countries, in which unfavorable working conditions compromise the provision of services offered in health facilities [25, 26].

Although Angola's Gross Domestic Product was around 70 billion USD in 2021, the country has the fourth highest unemployment rate and is the 48th poorest in the world. According to official data from the United Nations, four out of ten Angolans live in extreme poverty, especially in rural areas, where it is estimated that more than 87% of the inhabitants are in a situation of socioeconomic fragility, with a consumption level of 24.07 USD per month [27, 28]. This presents challenges in public services to meet the needs of the population, especially in the sectors of health and education. Education is a legitimate right of citizens because it ensures knowledge, reduces violence, combats poverty, helps in claiming other rights, and ensures the socioeconomic and cultural development of any society [29].

In our study, it was found that users with a monthly family income below 118.57 USD are more likely to adhere to the services offered by the public PHCUs, but they complain about the scarcity of necessary materials in the offices, examination laboratories, and, above all, the scarcity of drugs in the pharmacies because most of the time, they must resort to purchasing them in commercial pharmacies, which is a challenge for the low-income population and a limitation to accessing the treatment.

As for the care in the health units, it is predominantly dealt with by order of arrival (83.33%), which often implies that the user must arrive at dawn in order to receive medical care in the early hours of the day [25]. Since the PHCUs is the first contact of the user with the health system, the way the user is received has a great influence, taking into account that the user may be fragile and insecure when seeking health services.

Regarding geographical distribution, most professionals (54.17%) pointed to the distance between the health facilities and the user's residence as another factor that interferes with the adherence to the services offered in their health facilities. [30]. Meanwhile, users divided their opinions between being satisfied and not satisfied with this distance.

Users rated the service provided by both physicians and nurses of the health units as good, despite a lack of doctors. These findings disagree with those from the World Health Organization report [18], which states that participants showed dissatisfaction regarding the care received from these workers.

As in the study in Brazil [31], users classified as negative the fact of not having access to doctors in the facilities, since the number of users who never had contact with doctors in the facilities is quite high (72.34%), and this can be considered an important factor influencing the non-adherence of users to primary care facilities.

Despite user satisfaction with the performance of health professionals, their presence in Angola remains scarce. According to the World Bank report, by the year 2017, there were 0.2 doctors (among generalists and specialists) and 0.4 nurses for every 1,000 inhabitants [32] in Angola. Already in an article published in Novo Jornal, on January 26, 2021, it was reported by the Popular Movement for the Liberation of Angola (MPLA) that Angola has one doctor for 7,245 inhabitants and one nurse for 913 inhabitants [33]. However, the World Health Organization estimates that less than 2.3 health professionals (among doctors, nurses, and midwives) per 1,000 would be insufficient to achieve coverage of PHC needs [34].

Our study found a statistically significant association between the users' level of education and the services offered, as they rated as good the nurses' performance regarding diagnosis and medication (*P* = 0.0002), but the service to the user needs to be improved (*P* = 0.03). This shows that the higher the level of education, the greater the critical ability to evaluate the conditions of services offered.

Regarding the assessment of the quality of the health units, users characterized comfort as good and hygiene as poor. This finding disagrees with a study conducted in Mozambique, where users considered comfort and hygiene in the PHCU as acceptable, but in both studies, users considered the equipment used in the offices (furniture/clinic) as just acceptable [10].

In terms of expectations, users participating in our study reported the need for improvement in the opening hours of health units, in the way of caring, in the waiting time for consultation, and lack of necessary materials, among other aspects with greater predominance to the speed of care, and empathy and humanization on the part of professionals. Such data corroborate, in part, the results of a study conducted in Brazil [31], which shows that when health units have their services well structured, with a good organization in the reception process, speed of care, and resolution of problems, there is a production of good expectations and credibility regarding the care provided.

During this study, it was also found that there was a higher satisfaction of users to the services in one of the health units of the study (56%), whose high level of adherence and satisfaction may be related to the humanized care by health professionals, such as the warm reception, relaxed conversation during consultations, among other data (data not shown in the tables). The majority of professionals (69.17%) believe that the services provided focus on appreciation, respect, and dignity to the user's life, and the more the users feel safe and confident about the professionals, the higher the level of satisfaction and adherence to the services offered.

### Study limitations

The present study is limited in the fact that there is not enough literature published on the subject in Angola, a fact that motivated comparison with results from studies carried out in other countries.

## Conclusion

According to the results found and their discussions, the level of education been shown a crucial tool for the access and provision of primary health care in Luanda, which we conclude so health professionals with better academic preparation are in a better position to respond to the challenges that affect the health of the population, and Users helps create a participatory and demanding awareness of their rights, thus increasing their adherence to health services.

Socioeconomic conditions, such as the low income of users, together with the infrastructural and organizational decay of health facilities, are factors that severely affect the access, provision and use of health services offered in PHC units.

Therefore, for the improvement of the scenario presented in our research on the offer and access to primary health care, it is of great importance to know the opinion of citizens, both those who serve and those who are met, to seek realistic, inclusive strategies and,, mainly, satisfactory for everyone. Knowing that APS is the gateway to any national health system, if the needs of users are well served, it will result in reducing users seeking care at specialized and very high care levels.

## Supplementary Information

Additional file 1

Additional file 2

## Data Availability

The datasets generated and/or analyzed during the current study are not publicly available due the participants of this study did not giving written consent for their data to be shared publicly, but are available from the corresponding author on reasonable request.

## References

[1] Mitano F, Ventura CAA, Palha PF. Saúde e desenvolvimento na África Subsaariana: uma reflexão com enfoque em Moçambique. *Physis Revista de Saúde Coletiva, Rio de Janeiro*. 2016;26(3):901–15. Available at: 10.1590/S0103-73312016000300010.

[2] Angola: Lei nº 21-B/92 28 Agosto de 1992 (Lei de Bases do Sistema Nacional de Saúde, capítulo II, artigo 11º), alínea 1 e 2. In: Diário da República 1992; 34, I série. Available at: https://lex.ao/docs/assembleia-do-povo/1992/lei-n-o-21-b-92-de-28-de-agosto/.

[3] Angola: Política Nacional de Saúde. Decreto Presidencial nº. 262/10, de 24 de Novembro. Alínea 2.3.1, 2.3.2 e 2.3.3.1 sobre o Sistema Nacional de Saúde. 2010. Available at: https://lex.ao/docs/presidente-da-republica/2010/decreto-presidencial-n-o-262-10-de-24-de-novembro/.

[4] Angola: Regulamento Geral das Unidades Sanitárias do Serviço Nacional de Saúde. Decreto nº 54/03, de 5 de Agosto. Capítulo VII, alínea 1 sobre Atenção Primária à Saúde. 2002. Available at: https://lex.ao/docs/conselho-de-ministros/2003/decreto-n-o-54-03-de-05-de-agosto/.

[5] Cucubica, HEA: Análise do Projecto de Reforço dos Serviços de Saúde Materno Infantil na melhoria da qualidade dos Serviços da Rede de Atenção Primária na província de Luanda – Angola. [Dissertação de mestre, Escola Nacional de Saúde Pública] 2011. Available at: https://silo.tips/download/hirondina-esperana-de-armando-cucubica.

[6] Ministério da Saúde de Angola: Rede Sanitária Pública de Luanda. Luanda-Angola. Available at: https://www.ine.gov.ao/Arquivos/arquivosCarregados//Carregados/Publicacao_638386087956661449.pdf.

[7] Abrantes MJA. Qualidade e Satisfação: Opinião dos Utilizadores de Serviços de Saúde Hospitalares. *[Dissertação de mestre, Universidade de Coimbra]* 2012. Available at: https://estudogeral.sib.uc.pt.

[8] Afolabi MO, Faleye BA. Validação de construção de um instrumento para medir a satisfação do paciente com serviços farmacêuticos em hospitais nigerianos. *africano Saúde Ciências* Vol 12 Edição 4 de dezembro de. 2012;(4):538–44. Available at: 10.4314/ahs.v12i4.22.

[9] Hespanhol A. A Imagem dos Serviços de Saúde e dos Médicos de Família em Portugal. *Revista Portuguesa de Medicina Geral e Familiar.* 2005;21(2):185–91. Available at: 10.32385/rpmgf.v21i2.10123.

[10] Associação Zuwa: Satisfação dos Utentes dos Centros de Saúde da Cidade da Beira: Relatório Final de Pesquisa. Beira, Moçambique. 2012. Available at: http://www.iese.ac.mz.

[11] Giovanella L. Financiamento da Atenção Primária à Saúde. [Comunicação]. Debate sobre Financiamento da Atenção Primária à Saúde, Brasil. (2020, 17 de janeiro). Available at: https://www.abrasco.org.br.

[12] Luiz RR, Magnanini MM. A lógica da determinação do tamanho da investigação epidemiológica. *Cadernos Saúde Coletiva (Rio J.).* 2000;8:9–28. pág. 12. Available at: https://edisciplinas.usp.br.

[13] *Rosa,* V*SDS*: A satisfação Profissional dos Enfermeiros da Região Sanitária Santiago Norte, Cabo Verde. Estudo Transversal. 2019. (P: 50 e 54). Identifcation: https://repositorio.ul.pt›bitstream›12325 Tese. Available at: https://repositorio.ulisboa.pt/handle/10451/42139.

[14] Aroso M, Seabra P. Expectativas dos usuários na consulta nos Cuidados Primários de Saúde. *Revista Portuguesa de Medicina Geral e Familiar*. 2021;37(2):109–18. pág.114. Available at: 10.32385/rpmgf.v37i2.12929.

[15] Utino L, Birhanu B, Getachew N, Ereso BM. Qualidade percebida dos serviços médicos no ambulatório de hospitais públicos na zona de Dawro, sul da Etiópia. *Pesquisa de Serviços de Saúde BMC*. 2023;23:209. P. 4. Available at: 10.1186/s12913-023-09178-0.

[16] Instituto Nacional de Estatística: Resultados preliminares recenseamento geral da população e habitação – 2014, p. 48. Luanda, Angola. Available at: https://andine.ine.gov.ao/nada/index.php/catalog/3/related-materials.

[17] Gondima, APS, Andrade JT. Cuidado humanizado na atenção primária à saúde: demanda por serviços e atuação profissional na rede de atenção primária à saúde*.* Fortaleza, Ceará, Brasil*. Revista portuguesa de saúde pública.* 2014;32(1 ):61–68. Recuperado - www.elsevier.pt / rpsp0870–9025/$. Available at: 10.1016/j.rpsp.2014.01.002.

[18] Silveira TVL, Júnior PPP, Siman AG, Amaro MOF. Opinião dos enfermeiros sobre a utilização de indicadores de qualidade na assistência de enfermagem. Brasil. 2015. *Revista Gaúcha de Enfermagem*. Available at: 10.1590/1983-1447.2015.02.47702. 10.1590/1983-1447.2015.02.47702 26334413

[19] Viana KNR, Santos PU, Costa RM, Pardinho FGS, Sousa AQP, Quaresma FRP. Conhecimento dos profissionais da atenção primária acerca da rede de saúde. Palmas, Tocantins, Brasil. Revista Humanidades e Inovação v.4, n. 5 – 2017. **ISSN**: 2358–8322. Available at: https://revista.unitins.br.

[20] Dullie L, Meland E, Mildestvedt T, Hetlevik Ø, Gjesdal S. Qualidade dos cuidados primários na perspectiva dos pacientes: um estudo transversal da experiência dos pacientes ambulatoriais em unidades de saúde públicas na zona rural do Malawi. Pesquisa de serviços de saúde BMC. 2018;18:872. Available at: 10.1186/s12913-018-3701-X.

[21] Esperdião M, Trad LAB. Avaliação da Satisfação do Usuário. *Revista Ciência & Saúde Coletiva, *volume (1), 2005 pp. Available at: 10.1590/s1413-81232005000500031.

[22] Cantalino JLR, Scherer MDA, Soratto J, Schafer A, Anjos DSO. Satisfação do usuário em relação aos serviços de Atenção Primária à Saúde no Brasil. *Revista de. Saúde Pública.* 2021, pág.: 3. Available at: 10.11606/s1518-8787.20210550002533.

[23] Lei n.º 2/00 de 11 de Fevereiro de 2015: Lei Geral do Trabalho. art 3º.Angola. In: *Diário da República* 2015; 87, série I. Available at: http://www.consuladogeralangola-porto.pt/download/pt/lei-geral-do-trabalho-de-angolaa.pdf.

[24] Marin MJS, Moracvick MYAD, Marchioli M. Acesso aos serviços de saúde: Comparação da visão de profissionais e usuários da atenção básica. *Rev. enferm UERJ* , início/encerramento de. 2014;22(5):629–36. Rio de Janeiro, Brasil. Available at: 10.12957/reuej.2014.4238.

[25] Organização Mundial da Saúde/África: Sistemas de Saúde em África: Percepções e Perspectivas das Comunidades: Relatório de um Estudo Multipaíses . Brazzaville, República do Congo- ISBN: 978 929 034 0522. P: xv (33). 2012. Available at: https://www.afro.who.int/sites/default/files/2017-06/portuguese---health_systems_in_africa----2012.pdf.

[26] Fronteira I, Dussault G. Recursos humanos da saúde nos países africanos de língua oficial portuguesa: problemas idênticos, soluções transversais?. Brasil. *R. Eletr. de Com. Inf. Inov. Saúde*. Rio de Janeiro, v.4, n.1, p.78–85, mar., 2010. Fonte: www.reciis.cict.fiocruz.br. ISSN 1981–6278. Available at: 10.3395/reciis.v4i1.348pt.

[27] Fundo Monetário Internacional: FMI coloca Angola na posição 72 das maiores economias e 48 dos mais pobres. 2021. Available at: https://mercado.co.ao/economia/fmi-coloca-angola-na-posicao-72-das-maiores-economias-e-48-dos-mais-pobres-FY1067043.

[28] Instituto Nacional de Estatística: Índice de Pobreza Multidimensional de Angola-IPM-A. 2020. Available at: https://www.dw.com/pt-002/pobreza-em-angola-temos-um-pa%C3%ADs-que-vai-implodir/a-63459057.

[29] Centro de Estudos e Investigação Científica da Universidade Católica de Angola (CEIC-UCAN): Relatório Social Angolano 2019/20: pp:21–25;44–45. Available at: http://www.embajadadeangola.com/pdf/RELATORIO-ECONOMICO-2019-2020-VERSAO-FINALISSIMA-18-AGOSTO-2021Relatorio2021_01a264_FINAL.pdf.

[30] Oliveira MS, Artmann E. Regionalização dos Serviços de Saúde: Desafios para o Caso de Angola. ENSP/FIOCRUZ*Cad. Saúde Pública*. 2009;25(4):751–60. ISSN: 0102–311X. Available at: 10.1590/S0102-311X2009000400006. 10.1590/s0102-311x2009000400006 19347201

[31] Viegas APB, Carmo RF, Luz ZMP. Fatores que influenciam o acesso aos serviços de saúde na visão de profissionais e usuários de uma unidade básica de referência. Saúde Soc., volume 24, 2015, pp. Available at: 10.1590/S0104-12902015000100008.

[32] Banco Mundial (2017): Available at: https://data.worldbank.org/indicator/SH.MED.PHYS.ZS?locations=AO.

[33] Artigo Novo Jornal: Available at: https://novojornal.co.ao/sociedade/interior/saude-angola-tem-apenas-um-medico-para-7245-habitantes-e-um-enfermeiro-para-913-habitantes-100533.html.

[34] Organização Mundial de Saúde: Requisitos da força de trabalho em saúde para a cobertura universal de saúde e os Objectivos de Desenvolvimento Sustentável. (Observador de Recursos Humanos para a Saúde, 17). (2016) Available at: https://apps.who.int/iris/handle/10665/250330.

