# Supplementary material for: Factors influencing access to primary health care in Luanda, Angola
Source: BMC Health Serv Res. 2025 Feb 14;25:250. doi: 10.1186/s12913-024-12120-7 (PMC11827170; doi:10.1186/s12913-024-12120-7)
Supplement: Supplementary file 1 — Additional file 1 [file 12913_2024_12120_MOESM1_ESM.docx]

**Questionnaire for professionals**

**1. Age**________

**2. Sex:** Male (__) Female (__)

**3. Length of service**_____

**4. Level of education**

Primary (__)

Secondary (__)

Higher (__)

**5. Is your pay appropriate for the work you do?**

Yes (__) No (__)

**6. Work pace in the Health Unit:**

Intense (__) Fair (__) Calm (__**)**

**7. Has communication with users/patients been good?**

Very easy (__) Easy (__) Hard (__) They don't understand (__)

**8. Do you treat all patients equally?**

Yes (__) No (__) Sometimes (__)

**9. What is Primary healthcare for you?**

A network that provides basic health services to the population (__)

Takes care of those in need (__)

Primary level of care for patients (__)

Gateway to the Health System (__)

**10. Primary Healthcare network consists of:**

Health Center (__) Local healthcare (__)

Municipal Hospital (__) Provincial Hospital (__)

Specialist Hospital (__) Central Hospital (__)

**11. Do you feel comfortable working at a primary healthcare level?**

Yes (__) No (__)

**12. How do you rate the quantity of people who seek the health services offered?**

A lot (__) A little (__)

Reasonable (__) Exaggerated (__)

**13. What is behind the high demand in secondary and tertiary level hospitals?**

Lack of primary healthcare units facilities (__)

Poor performance of primary healthcare units (__)

Lack of doctors at primary healthcare unit (__)

Few professionals (__)

Near the user's home (__)

Exemption from payment for the services (__)

They feel better served (__)

Speed of service (__)

Lack of knowledge from users (__)

**14. The hospital care is provided on the basis of:**

Attendance form (__)

Time of arrival (__)

Appointment time (__)

Seriousness (__)

Convenience (__)

Alphabetical/numerical order (__)

**15. Why do users seek the services provided at this Healthcare Unit?**

It's close to home (__) It serves well (__)

No charge (__) Fast service (__)

**16. Why don't users seek the services provided at this Healthcare Unit?**

Not believing in your services (__)

Not well-received (__)

Delayed service (__)

It's far from home (__)

Prefer private units (__)

Prefer professionals who are their fellow-countryman (__)

They have no acquaintances in the center (__)

**17. What are the most used services on the primary level?**

Prenatal care (__) Wound Dressing (__)

Family Planning (__) Vaccinations (__)

Well child visit (__) Maternity (__)

Other (__)

**18. Is there a shortage of medicines and other materials at PHC centers?**

Yes (__) No (__) Sometimes (__)

**19. Is there a shortage of medicines and other materials at PHC units?**

Yes (__) No (__) Sometimes (__)

**20. Does the health unit offer an environment conducive to performing one's duties properly**?

Yes (__) No (__) Fair (__)

**21. Is the work provided with compassion?**

Yes (__) No (__) Sometimes based on bias (__)

**22. How do you consider the services offered at the Medical Center, considering their general characteristics?**

Very satisfying (__) Satisfying (__) Not very satisfying (__)

Irrelevant (__) Dissatisfying (__)

**23. Regarding priorities, what should be improved at the Center services?**

Quality of services

Business hours (__)

Sanitary facilities (__)

Information System (__)

Equipment (furniture/clinic) (__)

Other (__)

**THANK YOU!**

**Questionnaire for users**

**1. Age________**

**2. Gender** Male (__) Female (__)

**3. Level of education**

Illiterate (__)

Primary (__)

Secondary (__)

Higher (__)

**4. What is your professional situation?**

Student (__)

Public employee (__)

Works in private sector (__)

Retired (__)

Employed (__)

Self-employed (__)

Unemployed (__)

Other____________________.

**5. What is your family income?**

Less than 30 thousand Kwanzas (__) From 60 to 100 thousand Kwanzas (__)

From 30 to 60 thousand Kwanzas (__) More than 100 thousand Kwanzas (__)

**6. What is the reason for your medical appointment?**

Illness (__) Child's consultation (__)

Medical care (__) Wound dressing (__)

Exams (__) Vaccinations (__)

Prenatal appointment (__) Maternity (__)

Other______________________________________.

**7. How long did you wait for your appointment?**

On the same day (__)

One day (__)

Two days (__)

Two weeks (__)

Two to seven days (__)

Between two weeks and one month (__)

More than a month (__)

**8. How long does it take to get an appointment?**

Less than 15 minutes (__) More than 1 hour (__)

From 15 to 30 minutes (__) More than 2 others (__)

From 30 minutes to 1 hour (__)

**9. Are you well looked after by nurses?**

Yes (__) No (__)

**19. Do you agree with the opening hours of the Health Center?**

Yes (__) No (__)

**20. Indicate the time you think is most appropriate: _____________________.**

**21. How do you classify the distance you travel to get to the Health Center?**

Close (__) Very close (__) Far (__) Very far (__)

**22. Are you satisfied with this distance?**

Very satisfied (__) Satisfied (__)

Not very satisfied (__) Irrelevant (__)

Not at all satisfied (__)

**23. What reasons do you have for not going to the health center?**

Charging for certain services (__)

A lot of people die in these units (__)

Errors in test results (__)

They switch test results (__)

The person leaves sicker than they entered (__)

Lack of materials (__)

It is very crowded (__)

Lack of doctors (__)

**24. Reason(s) why you go to the center?**

It's close (__) It's free (__) The problem was not complex (__) Other (__)

**25. Reason(s) why you don't go to the center?**

It's too far (__)

He is mistreated (__)

Doesn't understand the nurses'/doctors' language (__)

Employees are lazy (__)

Side effects (negative) with the medication given (__)

There's a lot of violence (loitering around the healthcare center) (__)

**26. Are you satisfied with the environment in the healthcare center?**

Yes (__) No (__)

**27. What makes you use another health service?**

There are no doctors at the healthcare center (__)

Poor service in the center (__)

When you have a serious problem (__)

No difficulties (__)

**28. What is your opinion of the health center?**

Good (__) Bad (__)

So and so (__) Very bad (__)

**29. What is your main difficulty in utilizing the healthcare center?**

There are no doctors (__)

Time-consuming service (__)

Distance (__)

Service is bad (__)

There are no difficulties (__)

**30. How do you rate the admission process (reception) at the Health Center?**

Very good (__) Good (__) Acceptable (__)

Sufficient (__) Bad (__) Very Bad (__)

**31. How do you rate the performance of doctors in relation to:**

**Diagnosis and medication**

Very good (__) Good (__) Acceptable (__) Sufficient (__) Bad (__) Very Bad (__)

**Performance of duties**

Very good (__) Good (__) Acceptable (__) Sufficient (__) Bad (__) Very Bad (__)

**Friendliness and availability**

Very good (__) Good (__) Acceptable (__) Sufficient (__) Bad (__) Very Bad (__)

**32. How do you rate the performance of nurses in relation to:**

**Diagnosis and medication**

Very good (__) Good (__) Acceptable (__) Sufficient (__) Bad (__) Very Bad (__)

**Performance of duties**

Very good (__) Good (__) Acceptable (__) Sufficient (__) Bad (__) Very Bad (__)

**Friendliness and availability**

Very good (__) Good (__) Acceptable (__) Sufficient (__) Bad (__) Very Bad (__)

**33. How do you rate the performance of other employees in relation to you?**

**Performance of duties**

Very good (__) Good (__) Acceptable (__) Sufficient (__) Bad (__) Very Bad (__)

**Friendliness and availability**

Very good (__) Good (__) Acceptable (__) Sufficient (__) Bad (__) Very Bad (__)

**34. During your consultation, did you carry out diagnostic, treatment and therapeutic tests (analysis, serum, vaccine, etc.)?**

Yes (__) No (__)

Other______________________.

**35. How would you rate the performance of the health technicians during the examination?**

Very good (__) Good (__) Acceptable (__) Sufficient (__) Bad (__) Very Bad (__)

**36. How would you rate the following aspects of the examinations carried out?**

**Waiting area**

Very good (__) Good (__) Acceptable (__) Sufficient (__) Bad (__) Very Bad (__)

**Waiting time to start the assistance**

Very good (__) Good (__) Acceptable (__) Sufficient (__) Bad (__) Very Bad (__)

**Waiting time for results**

Very good (__) Good (__) Acceptable (__) Sufficient (__) Bad (__) Very Bad (__)

**37. Did you use any of the following services at the Health Center during your consultation?**

**Yes No**

Volunteer services (__) (__)

Social work (__) (__)

Pharmacy (__) (__)

**Others_________________________.**

**38. How do you rate your satisfaction with the following services?**

**Volunteer services**

Very good (__) Good (__) Acceptable (__) Sufficient (__) Bad (__) Very Bad (__)

**Social services**

Very good (__) Good (__) Acceptable (__) Sufficient (__) Bad (__) Very Bad (__)

**Pharmacy**

Very good (__) Good (__) Acceptable (__) Sufficient (__) Bad (__) Very Bad (__)

**Other_________________________.**

**39. How do you rate the quality of the Health Center's facilities in terms of:**

**Indication/signposting of services:**

Very good (__) Good (__) Acceptable (__) Sufficient (__) Bad (__) Very Bad (__)

**Means of support for the disabled**

Very good (__) Good (__) Acceptable (__) Sufficient (__) Bad (__) Very Bad (__)

**Waiting room**

Very good (__) Good (__) Acceptable (__) Sufficient (__) Bad (__) Very Bad (__)

**Consultation rooms**

Very good (__) Good (__) Acceptable (__) Sufficient (__) Bad (__) Very Bad (__)

**Equipment (furniture and clinical)**

Very good (__) Good (__) Acceptable (__) Sufficient (__) Bad (__) Very Bad (__)

**Physical environment (noise, light, air conditioning, garden, decoration space)**

Very good (__) Good (__) Acceptable (__) Sufficient (__) Bad (__) Very Bad (__)

**Sanitary facilities**

Very good (__) Good (__) Acceptable (__) Sufficient (__) Bad (__) Very Bad (__)

**Hygiene or cleanliness**

Very good (__) Good (__) Acceptable (__) Sufficient (__) Bad (__) Very Bad (__)

**Privacy**

Very good (__) Good (__) Acceptable (__) Sufficient (__) Bad (__) Very Bad (__)

**Comfort**

Very good (__) Good (__) Acceptable (__) Sufficient (__) Bad (__) Very Bad (__)

**40. In general, how satisfied or dissatisfied are you with the services provided by the health center?**

Very satisfied (__) Not very satisfied (__)

Satisfied (__) Irrelevant (__)

Not at all satisfied (__)

**41. Taking into account the services provided by the health center and its general characteristics, how do you consider them?**

Very good (__) Good (__) Acceptable (__) Sufficient (__) Bad (__) Very Bad (__)

**42. In terms of expectations, in your opinion, what do you think should be improved at the health center as a priority {name three (3) aspects}:**

The quality of the health center's services (__)

The opening hours of the health center (__)

The form of service (__)

Waiting time for an appointment (__)

The lack of necessary materials (__)

Performance of administrative staff (__)

Performance of medical and technical staff (__)

Facilities (__)

Information systems (__)

**Others_________________________________.**

**THANK YOU FOR YOUR PARTICIPATION!**
