# Supplementary material for: Factors influencing access to primary health care in Luanda, Angola
Source: BMC Health Serv Res. 2025 Feb 14;25:250. doi: 10.1186/s12913-024-12120-7 (PMC11827170; doi:10.1186/s12913-024-12120-7)
Supplement: Supplementary file 2 — Additional file 2 [file 12913_2024_12120_MOESM2_ESM.pdf]

GET PUBLISHED

CERTIFICATE OF ENGLISH EDITING

This is to certify that the manuscript listed below has been proofread and edited by a native English speaker, specialised in proofreading academic and scientific work.

Manuscript:

“FACTORS INFLUENCING ACCESS TO PRIMARY HEALTH CARE IN LUANDA, ANGOLA ”

Corresponding Author:

Marli Stela Santana

To verify:

admin@getpublished.com.br or WhatsApp +55 (48) 99112 1454

www.getpublished.com.br

Registered Brazilian company number: CNPJ 32.235.765/0001-05
